# Comparative proteomics reveals distinct functions and localization of invasive *Entamoeba histolytica* and non-invasive *Entamoeba moshkovskii* proteins

**DOI:** 10.1186/s13071-026-07524-9

**Published:** 2026-06-24

**Authors:** Somphob Leetachewa, Narumol Khomkhum, Sittiruk Roytrakul, Onrapak Reamtong, Porntip Chavalitshewinkoon-Petmitr, Saengduen Moonsom

**Affiliations:** 1 Institute of Molecular Biosciences, Mahidol University, Nakhon Pathom, 73170 Thailand; 2 Department of Protozoology, Faculty of Tropical Medicine, Mahidol University, Ratchathewi, Bangkok, 10400 Thailand; 3 National Center for Genetic Engineering and Biotechnology, National Science and Technology Development Agency, 113 Thailand Science Park, Klongnueng, Klong Luang, Pathumthani, 12120 Thailand; 4 Department of Molecular Tropical Medicine and Genetics, Faculty of Tropical Medicine, Mahidol University, Bangkok, Thailand; 5 Mahidol Oxford Tropical Medicine Research Unit, Faculty of Tropical Medicine, Mahidol University, Bangkok, Thailand

**Keywords:** *Entamoeba histolytica*, *Entamoeba moshkovskii*, Comparative proteomics, GeLC–MS/MS, Immunolocalization

## Abstract

**Background:**

*Entamoeba histolytica* is a pathogenic protozoan accountable for amoebiasis, while *Entamoeba moshkovskii* is considered non-invasive. Despite morphological similarity, the molecular mechanisms underlying their different pathogenicity remain largely undefined.

**Methods:**

Trophozoite proteins from axenic cultures of *E. histolytica* and *E. moshkovskii* were separated and identified using GeLC–MS/MS, and classified using Gene Ontology. Selected and differentially expressed proteins were validated by peptide-specific antibody production, ELISA, and immunofluorescence to determine cellular localization.

**Results and discussion:**

A total of 1,077 and 1,201 proteins were identified from *E. histolytica* and *E. moshkovskii*, respectively. The 801 of *Entamoeba* common proteins included kinases, GTPase-activating proteins, and heat shock proteins, reflecting conserved cellular processes. *E. histolytica*-unique proteins involved in nitrogen compound metabolism, vesicle-mediated transport, and catalytic activities, whereas *E. moshkovskii* proteins were related to lipid metabolism and environmental resilience. Subcellular localization revealed species-specific distribution of MmpL and AIG1-family proteins, suggesting potential roles in pathogenicity and host-immune response. A large proportion of hypothetical proteins was identified, highlighting gaps and opportunities for future study.

**Conclusions:**

Our study highlights conserved and divergent functions and cellular locations of *Entamoeba* species-specific proteins as insights for distinct pathogenicity and adaptation. MmpL and AIG1 proteins were proposed as potential targets for further diagnostic and therapeutic development.

**Graphical Abstract:**

**Figure:**
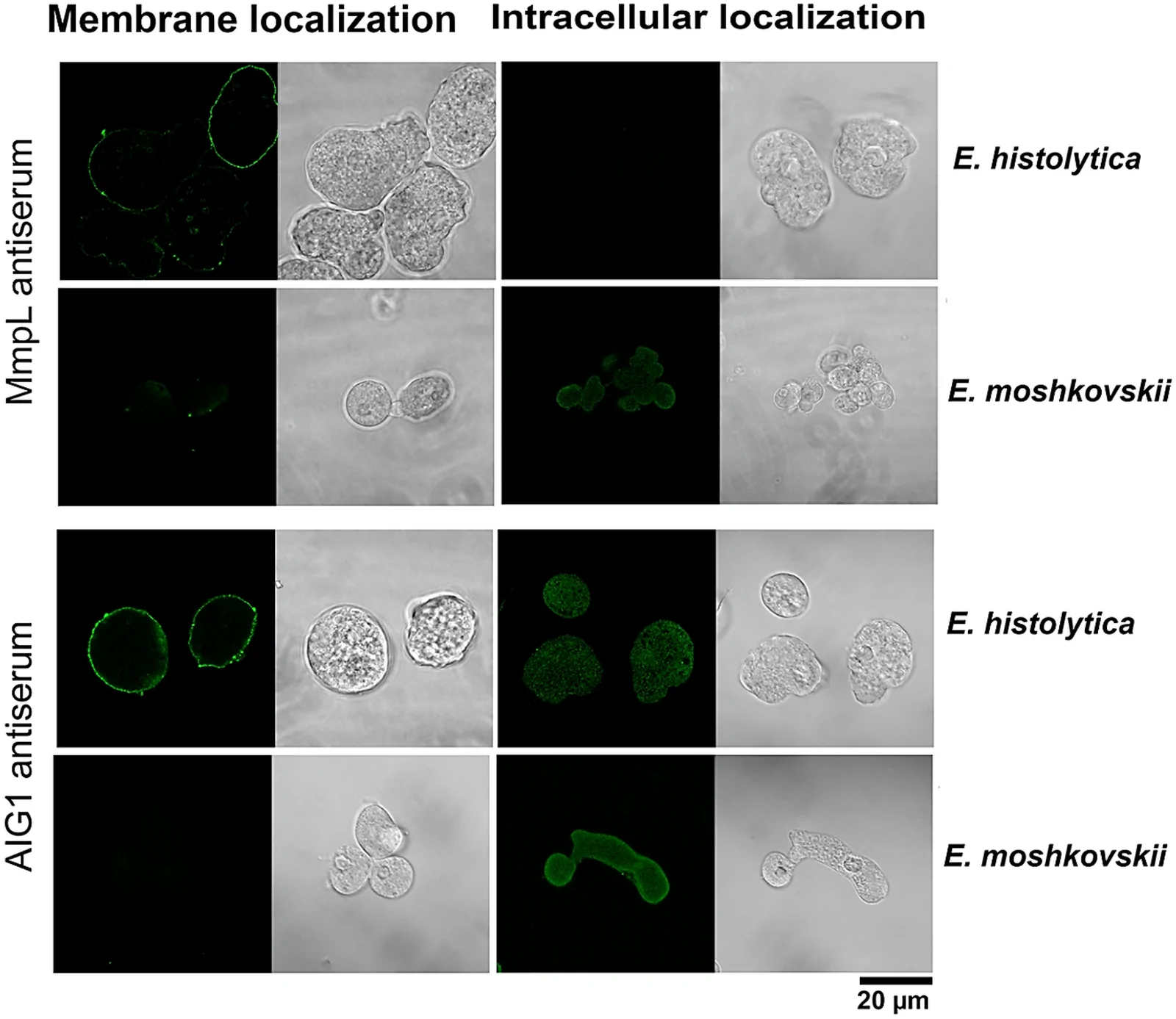

**Supplementary Information:**

The online version contains supplementary material available at https://doi.org/10.1186/s13071-026-07524-9.

## Background

*E. histolytica, E. dispar, E. moshkovskii, E. coli, E. hartmanni, E. polecki, and E. bangladeshi* are protozoa inhabiting at human intestine. *E. dispar* is the most prevalent (49%) followed by *E. histolytica* (32%) and *E. moshkovskii* (10%) [1]*.* Most of *E.* histolytica infections are asymptomatic, while symptomatic cases rank from bloody diarrhea or dysentery, colitis, and amoebic liver abscess, resulting in approximately 55,000 of the global deaths annually [2]. *E. moshkovskii* has been associated with diarrhea in infants, whereas *E. dispar* is considered non-pathogenic [3]. Reliable diagnosis is challenging because these species are morphologically indistinguishable by the gold standard microscopy, leading to misdiagnosis and suboptimal treatment and emergence of anti-microbial resistance [4, 5].

The life cycle of *Entamoeba* involves cyst and trophozoite which are an infective and invasive stage, respectively. The previous comparative genomics has revealed significantly diversity in gene families of *E. moshkovskii*, *E. histolytica*, *E. dispar* and *E. invadens* [6]. Mass spectrometry-based proteomics has been widely applied as a powerful approach for discovering proteins involved in pathogenesis, disease progression, and as potential therapeutic or vaccine targets [7, 8]. Previous studies have identified several trophozoite membrane proteins, including alcohol dehydrogenase 3, disulfide isomerase, enolases, heat shock proteins, malate dehydrogenase, triose phosphate isomerase, superoxide dismutase and thioredoxin as virulence factors of the *E. histolytica* [9–12]. Other proteins such as pyruvate phosphate dikinase, putative acetyl-CoA synthetase, 170-kDa galactose adhesin have been shown to play roles in intestinal colonization and host immune evasion [13]. While the *E. histolytica* specific 14-kDa tyrosine kinase was identified as an antigenic protein from ALA patients [14]. While encystation mechanisms are well characterized in *E. invadens*, proteomic evidence suggests roles for proteolysis, redox homeostasis, and stress responses in *E. histolytica* encystation [15]. Nonetheless, the functional proteomes of *E. moshkovskii* and another human *Entamoeba* remain rarely characterized. The current study aims to provide comparative proteomic profiling and functions of *E. moshkovskii* and *E. histolytica.* Cellular localizations of selected proteins were assessed to better understand in species-specific pathogenic mechanism of these two closely related *Entamoeba* species.

## Methods

### Axenic *Entamoeba* cultivation and protein preparation

*E. histolytica* (HM1: IMSS) and *E. moshkovskii* (Laredo) were provided by Professor Tomoyoshi Nozaki, Department of Biomedical Chemistry, Graduate School of Medicine, University of Tokyo. Trophozoite cells were cultured axenically in BIS medium supplemented with 15% of adult bovine serum, following established protocols [16]. Minimal-cell subpassage were performed (one or two cycles) to minimize loss of virulence factors. After harvesting by centrifugation at 200 × g for three minutes, cells were washed with cold PBS (pH 7.4). Total cellular proteins were extracted using the Mammalian Protein Extraction Reagent (Thermo Fisher Scientific, Waltham, MA, USA) supplemented with a protease inhibitor cocktail and 150 mM of the cysteine-proteinase inhibitor E-64 (Sigma-Aldrich, MO, USA). Extraction followed the manufacturer’s instructions and the protocol of Khomkhum et al. [17]. SDS was added to a final concentration of 0.1% for hydrophobic protein solubilization. The excess SDS was subsequently removed by deoxycholate (0.15%)—trichloroacetic acid (72%) precipitation and protein quantified by the modified Lowry assay [18].

### SDS–polyacrylamide gel electrophoresis and tandem mass spectrometry (GeLC–MS/MS)

One hundred micrograms of trophozoite proteins were prepared three independent *Entamoeba* cultures in a loading buffer containing dithiothreitol, sodium dodecyl sulfate (SDS), Tris–HCl, glycerol and bromophenol blue dye. Proteins were heated at 90 °C for 5 min and separated (100 µg per lane) by 12% SDS-PAGE (Bio-Rad Laboratories, Benicia, CA, USA), stained with Coomassie Brilliant blue G and scanned with a GS-710 scanner (Bio-Rad Laboratories). Each protein lane was cut into 15 equal segments (approximately 1 mm^3^ each) using a cutting guide to systematically fractionate the full molecular weight range resolved by SDS-PAGE. Corresponding gel sections from the triplicate lanes were processed in parallel for dehydration, reduction, alkylation, overnight tryptic digestion [19]. The tryptic peptides were extracted from the gel using 0.1% formic acid containing 50% acetonitrile. Peptides corresponding to each gel section were combined across the triplicate preparations. LC–MS/MS analysis of pooled tryptic peptides was performed using a Waters SYNAPT HDMS system equipped with Waters nano ACQUITY UPLC system (Waters Corp., Manchester, UK). Peptides (100 ng) were separated on a 20 cm × 75 µm reverse-phase column packed with 1.7-µm BEH C18 (Waters) using a linear gradient of 2–40% acetonitrile at 350 nL/minute for 60 min. The column was washed with 80% and re-equilibration to with 2% acetonitrile for 15 min. Mass spectra of eluted peptides were acquired by electrospray ionization and calibrated using [Glu1] fibrinopeptide B as an external mass reference to ensure consistent mass accuracy across runs. Tryptic digested bovine serum albumin was run periodically as a QC standard to monitor ionization efficiency and peptide identification consistency. Solvent-blank runs were performed every five samples to minimize carryover and preserve data integrity across batches.

### Data mining, quantitation and protein identification

Label-free protein quantitation was performed using DeCyder MS Differential Analysis version 2.0 [20]. GeLC–MS raw data were processed using the PepDetect module in the DeCyder MS Differential Analysis for automated peptide detection, charge state assignment, and label-free quantitation based on ion signal intensities. Data were normalized to reduce technical variation, and quantitative comparisons were performed using integrated peptide intensities. Statistical significance between groups was evaluated using Student’s *t*-test, with a significance threshold of *P* < 0.05. MS/MS data were searched using Mascot against the NCBI *Entamoeba* database, including a decoy database, and identifications were filtered at a 1% false discovery rate at both peptide and protein levels. Search parameters were as follows: taxonomy, Entamoeba; enzyme, trypsin with up to one missed cleavage; variable modifications, carbamidomethyl (C) and oxidation (M); mass type, monoisotopic; protein mass, unrestricted; peptide mass tolerance, 1.2 Da; fragment mass tolerance, ± 0.6 Da; and charge states, 1 + , 2 + , and 3 + . Proteins were considered confidently identified when at least two peptides matched with Mascot scores corresponding to *P* < 0.05. Relative expression of a protein was then calculated as log_2_ [21]. Folds of relative-protein expression is 2^*n*^, when n is protein log_2_ of the relative-protein expression value appeared in a table.

### Bioinformatics analysis and protein classification

Identified proteins were analyzed using BLASTp [22] of NCBI and confirmed for description or annotation of species-specific proteins using AmoebaDB [23]. Visualization and proteomic comparison were performed with BioVenn [24]. Functional categories were assigned using Gene Ontology (GO) [25] with a significance threshold of *P* < 0.05. Proteins without functional annotation were labeled as “uncharacterized”. Functional classification was re-checked for updated and comprehensive annotation by AmoebaDB at the time of analysis in February 2026.

### Polyclonal antibody production to GeLC–MS/MS identified proteins

Proteins were selected for experimental validation based on their high relative expression, availability of complete amino-acid sequences and known or putative biological function. Peptides were synthesized (GenScript Biotech Corporation, Piscataway, NJ, USA) based on de novo or aligned sequences. These sequences include RVNAYFENKNDNYYITLGFD of *E. invadens* IP1 putative protein serine/threonine kinase (XP_004258059.1), VIVRENTVLTNKTQEISE of *E. invadens* IP1 Mycobacterial membrane protein large or MmpL (XP_004257468.1) and NIVQEEGGING of *E. histolytica* HM-1: IMSS-B Androgen-induced gene 1 protein; AIG1 (XP_648471.1). Synthetic peptides and *Entamoeba* cell-lysate proteins were mixed with alum adjuvant (Thermo Scientific, Rockford, IL, USA) at 3:1 ratio of peptide:alum. Protein/peptide and alum mixture was injected into mice at 100 μg per dose, intraperitoneally and with 2 week interval for six doses. Serum antibody titers were measured by Enzyme Linked Immunosorbent Assay (ELISA) and dot blotting with *Entamoeba*-cell lysate proteins and the synthetic peptides. Three of independent experiments were performed. 

### Immunofluorescence trophozoite staining with specific antibodies

Trophozoite cells were fixed on glass slides with 3.7% paraformaldehyde. The cells were permeabilized with 1% BSA-PBS containing 0.2% Triton X-100 and reacted with immunized or pre-immune sera (1:1,000) at 37 °C for 1 h. FITC-conjugated anti-mouse IgG (at 1:60 dilution) was used for detection, and slides were examined by confocal microscopy (LSM700, Carl Zeiss, Geneva, Germany). All experiments were performed in triplicate with established protocols [17].

## Results

### Comparative proteomic profiling of *E. histolytica* and *E. moshkovskii* trophozoites

**Fig. 1:**
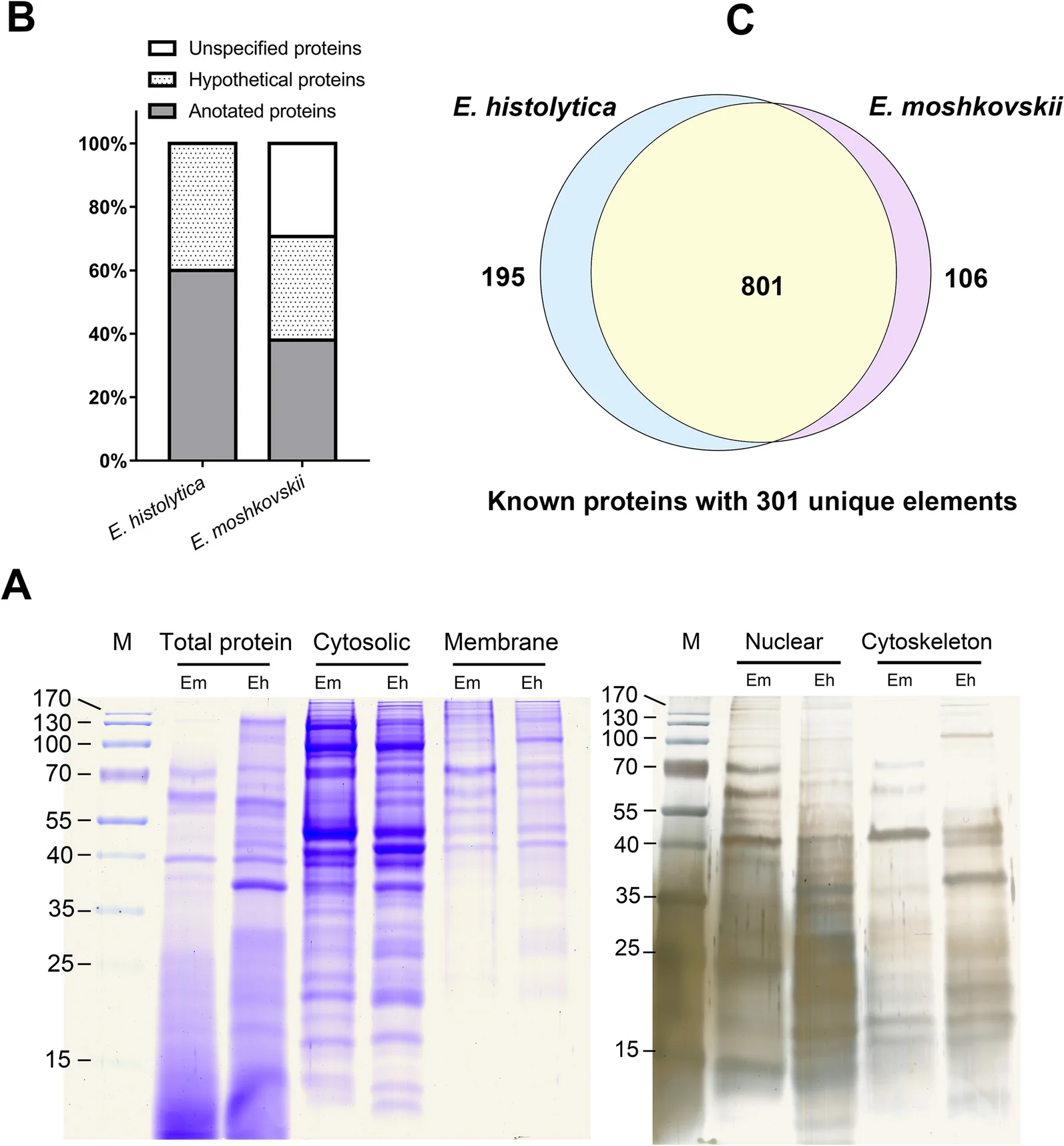
Comparative protein profiles of *E. histolytica* and *E. moshkovskii*. Total cell-lysate proteins, cellular compartments isolated from *E. histolytica* (Eh) and *E. moshkovskii* (Em) separated by SDS-PAGE and stained with Coomassie brilliant blue and silver nitrate (**A**). Percentage of annotated, hypothetical and unspecified proteins identified by LC–MS/MS for each species (**B**). Comparison of known proteins using Venn diagram (**C**)

Proteomic analysis identified a total of 1,077 proteins were identified in *E. histolytica* trophozoites and 1,201 proteins in *E. moshkovskii* trophozoites using GeLC–MS/MS (Additional file: Table S1; Fig. 1A). Among them, 801 proteins were conserved in both species, while 195 and 106 proteins were unique (down- or up-regulated) in *E. histolytica* and *E. moshkovskii*, respectively (Fig. 1C). Functional classification by GO demonstrated that 60% of *E. histolytica* and 38% of *E. moshkovskii* proteins had known functions. The remaining proteins were classified as hypothetical or uncharacterized, particularly in *E. moshkovskii* (Fig. 1B).

### Functional classification of *Entamoeba* common proteins

**Fig. 2:**
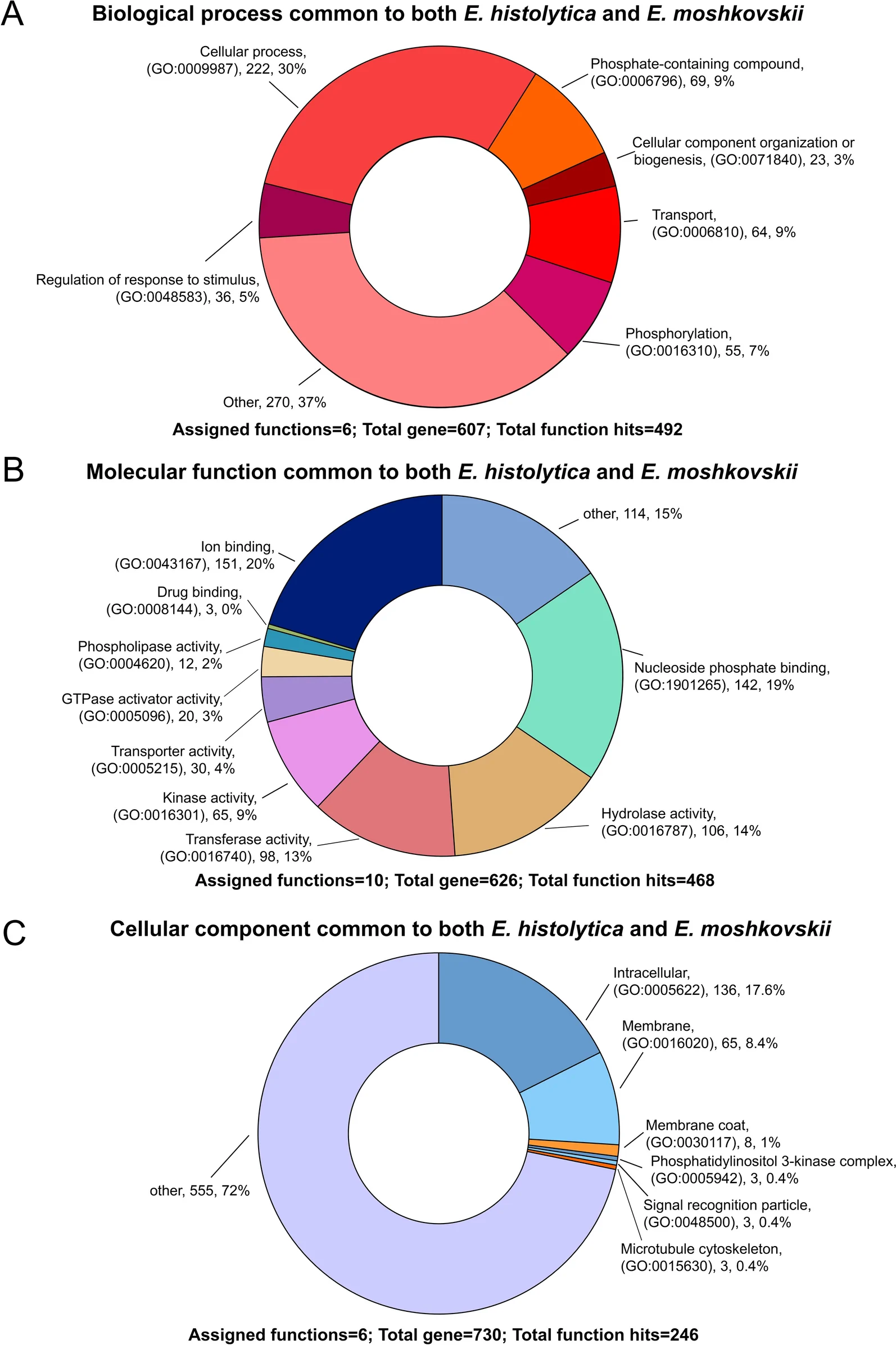
Functional categories shared between *E. histolytica* and *E. moshkovskii.* Pie charts display proportions that are common between *E. histolytica* and *E. moshkovskii* and assigned to biological process (**A**), molecular function (**B**) and cellular component (**C**)

Analysis of 801 shared proteins (Additional file: Table S2) provided a range of biological processes, with 222 (30%) involved in cellular processes, 64 (9%) in transport, 69 (9%) in phosphate-containing compounds, 55 (7%) in phosphorylation, 36 (5%) in regulation of response to stimuli, and 23 (3%) in cellular component organization (Fig. 2A). Regarding molecular function, 329 proteins (41%) had catalytic activity, 296 (39%) were involved in binding, and 30 (4%) participated in transporter activity (Fig. 2B). Analysis of subcellular localization showed that 555 out of 730 proteins (72%) had unknown localization, while 136 (17.6%) were intracellular and 65 (8.4%) were membrane-associated (Fig. 2C). Multiple families were represented within species common proteins, including serine/threonine/tyrosine kinases, Rab/Ran/Ras GTPase-activating proteins, and heat shock proteins (Table 1). Protein categories such as leucine-rich repeat (BspA) proteins, RNA recognition motif proteins, Sec1 family proteins, and WD domain-containing proteins were also identified with varying representation between two *Entamoeba* species.

**Table 1:** Protein shared between *E. histolytica* and *E. moshkovskii*

Name	Number of protein IDs assigned	
	*E. histolytica*	*E. moshkovskii*
Putative serine/threonine/tyrosine kinase	48	23
Rab/Ran/Ras GTPase-activating proteins	38	28
Leucine rich repeat protein, BspA family	0*	5
WD domain containing protein	10	9
Putative Ras-guanine nucleotide exchange factor	8	3
HEAT repeat protein	7	3
Putative heat shock protein 70	6	1
Putative acetyl-CoA synthetase	5	2
Putative sucrose transporter	3	1
Putative myotubularin	4	2
Putative adenosine-triphosphate binding cassette transporter (ABC) transporter	3	1
Putative phosphatidylinositol 3-kinase (PI3K)	3	1
RNA recognition motif containing protein	2	4
Putative helicase	1	2
Pumilio family RNA-binding protein	1	2
Sec1 family protein	2	3
TPR repeat protein	1	2
Ubiquitin-conjugating enzyme family protein	1	2
Putative villidin	1	2

^*^15 ID assigned for leucine rich repeat protein and BSpA (searched in 2023) were reclassified by GO as uncharacterized proteins by analysis in February 2026

### Differential expression of species-specific proteins

A total of 195 proteins were uniquely expressed in *E. histolytica* trophozoites (Additional file: Table S3; Fig. 1C). The top ten of the high-expression proteins included a putative 70-kDa heat shock protein, phospholipid-translocating P-type ATPase, Rab-family GTPase, protein kinase domain–containing protein, aminotransferase, PI3K, DEAD/DEAH box helicase, AIG1-family protein, signal recognition particle protein SRP54, and choline/ethanolamine kinase (Table 2, upper panel). These proteins exhibited expression levels between 70- and 20-fold higher than in *E. moshkovskii*. In contrast, 106 proteins were *E. moshkovskii* unique (Additional file: Table S4). The top-ten proteins included Sec1 family protein, elongation factor 1-alpha 1, putative mitochondrial carrier protein, phospholipase B, seryl-tRNA synthetase, Rab family GTPase, actin, signal recognition particle receptor alpha subunit, steroidogenic acute regulatory (START) domain-containing protein, and Ran binding protein (Table 2, lower panel). These proteins showed 63- to 14-fold higher expression levels compared to *E. histolytica*.

**Table 2:** Top ten proteins with highest relative expression identified from *E. histolytica* and *E. moshkovskii*

*E. histolytica* gene ID	Description of AmoebaDB	Log_2_ folds of relative protein expression	De novo peptide sequence
The top ten of *E. histolytica* proteins			
EHI_064470	Putative 70-kDa heat shock protein	+ 6.12	IIKLDLFR
EHI_174280	Putative phospholipid-translocating P-type ATPase	+ 5.98	GADNIMIER
EHI_026420	Rab-family GTPase	+ 5.89	SSIVLR
EHI_084670	Putative aminotransferase,	+ 5.87	MINICKK
EHI_109700	Protein kinase domain containing protein	+ 5.72	CTSCDTTSEFAILK
EHI_013980	Putative PI3K	+ 5.25	NQIDLCKK
EHI_014080	Putative DEAD/DEAH containing helicase	+ 4.84	ERVAILR
EHI_119040	Putative AIG1-family protein	+ 4.51	MQEEGGINLE
EHI_004750	Putative signal recognition particle protein SRP54	+ 4.42	NMLNDEKR
EHI_152340	Putative choline/ethanolamine kinase	+ 4.36	KETQQR
The top ten of *E. moshkovskii* proteins			
EMO_119350	Sec1 family protein	−5.98	GFHPLITR
EMO_123750	Elongation factor 1-alpha 1	−5.74	IVPTKPLCVEEFAK
EMO_063880	Putative mitochondrial carrier protein	−5.53	TVVAPLDR
EMO_016580	Putative phospholipase B	−5.07	DHMNNQR
EMO_024820	Putative seryl-tRNA synthetase	−4.59	GFLPIQTPFFMRR
EMO_043380	Rab family GTPase	−4.59	TLFNALDSK
EMO_049420	Putative actin	−4.51	SYELPDGQVISIGNER
EMO_013470	Putative signal recognition particle receptor alpha subunit	−4.50	MQGNDALMK
EMO_062540	Steroidogenic acute regulatory protein (START) domain containing protein	−4.42	LKTMALK
EMO_033190	Ran binding protein, putative	−4.28	MEEYPK

Values are shown as log_2_ of relative-protein expression (see Methods for calculation) between *E. histolytica* and *E. moshkovskii*. Symbols “ + ” and “ − ” denote higher and lower in relative-protein expression in *E. histolytica*, respectively. Full quantitative values, statistical criteria, and protein identifiers are provided in additional files of the supplementary tables

### Functional categorization and biological roles of unique proteins

**Fig. 3:**
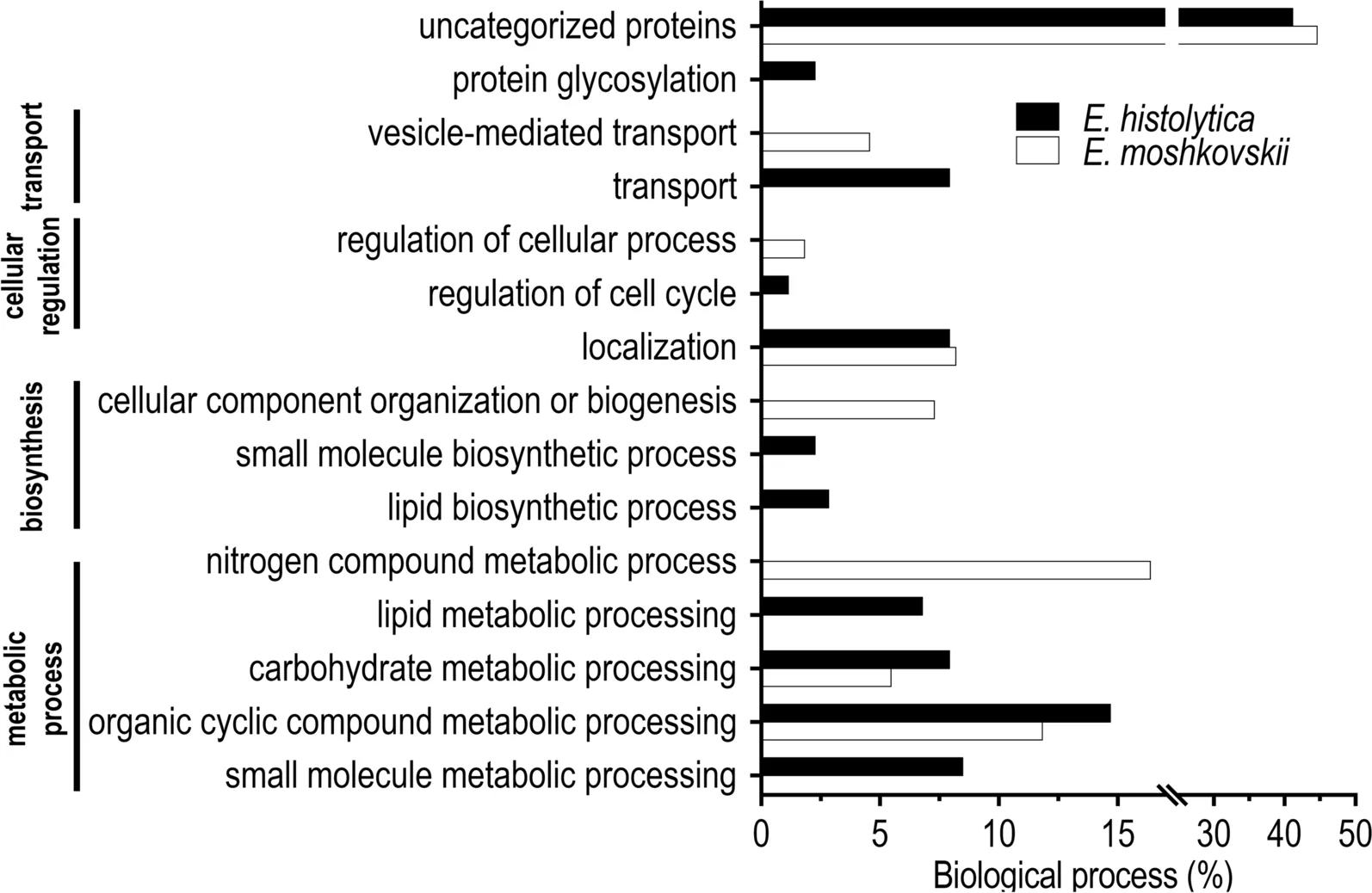
Proportions and functions in biological processes of the proteins that are unique to *E. histolytica* and *E. moshkovskii*

The unique proteins were analyzed further for their functions and were assigned to four major biological processes: biosynthesis and metabolism of lipids, carbohydrates, and small molecules; transport; and cellular regulation (Fig. 3). A substantial proportion (40–45%) of the unique proteins of both species were uncategorized. Among functionally classified proteins, metabolic processes accounted for nearly 40%, transport and localization 16%, and cellular regulation a smaller but significant fraction. In *E. histolytica*, approximately 20% of unique proteins were linked to nitrogen compound metabolism, and 5–8% were associated with vesicle-mediated transport and cellular component biogenesis. *E. moshkovskii* unique proteins were enriched with those involved in lipid and small molecule metabolism (18%), protein glycosylation, and biosynthetic processes.

### Molecular function of unique proteins

**Fig. 4:**
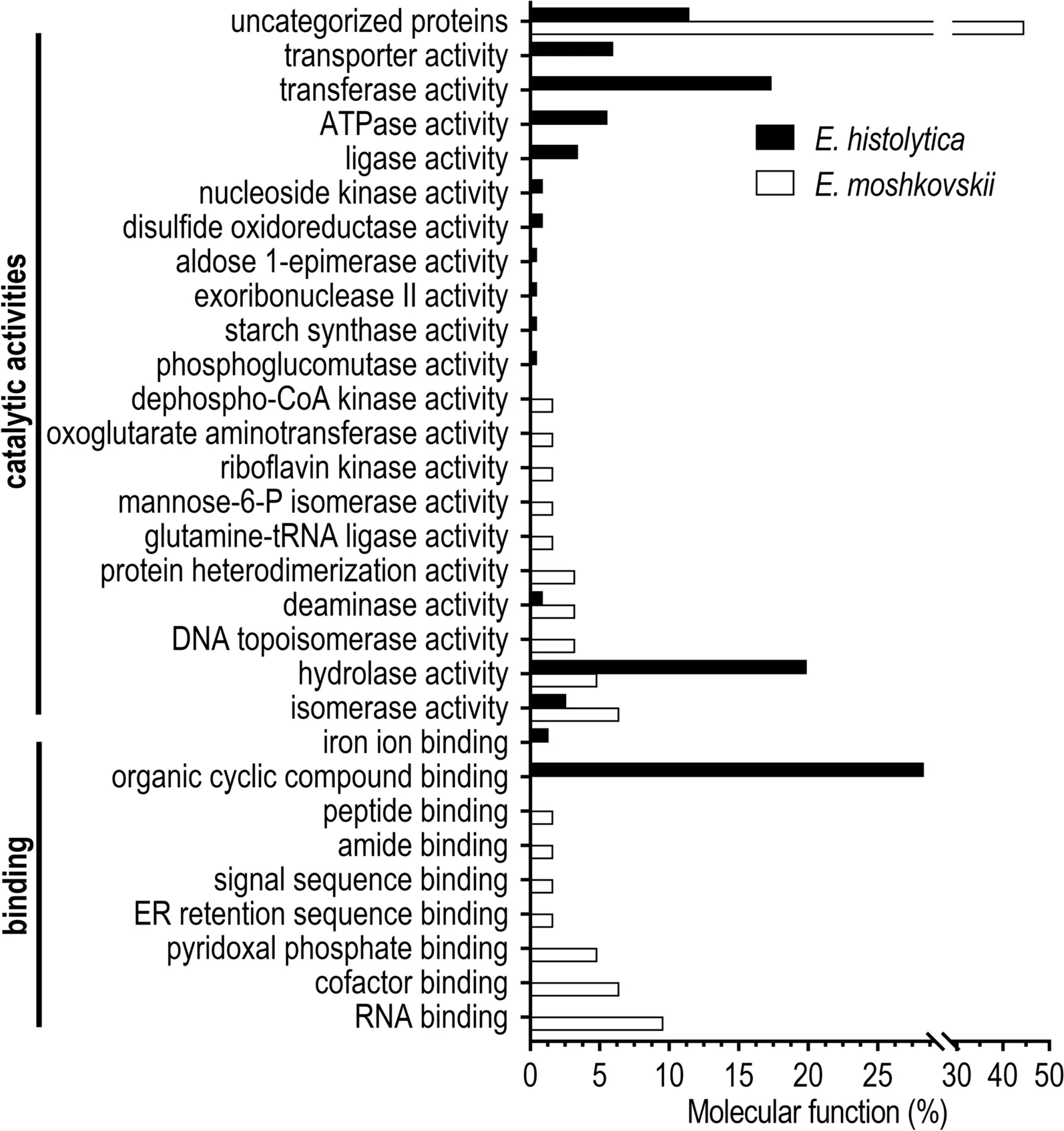
Proportions and molecular functions of the proteins that are unique to *E. histolytica* and *E. moshkovskii*

Molecular function analysis revealed that *E. histolytica* specific proteins include a greater diversity of catalytic enzymes such as transferases and ATPases, as well as proteins involved in binding to organic cyclic compounds and iron ions (Fig. 4). In contrast, most of *E. moshkovskii* unique proteins were uncategorized, with only small proportions assigned to catalytic or binding functions.

### Protein identification and subcellular localization

**Fig. 5:**
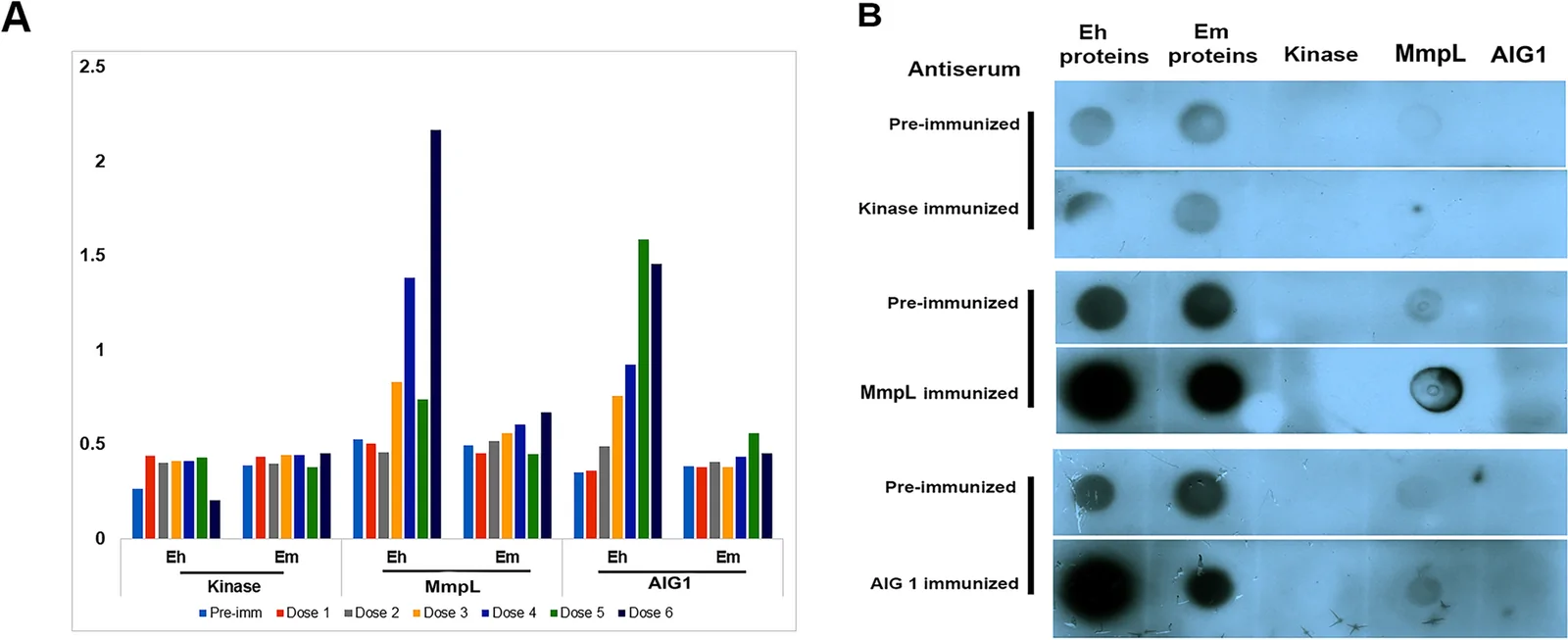
Reactivity between specific antibody and the identified proteins of *E. histolytica* and *E. moshkovskii* cells. Expression of GeLC- MS/MS identified proteins; kinase, MmpL and AIG1 were determined by ELISA with antisera obtained from mice immunized with the corresponding synthetic peptides for six doses (**A**). Binding specificity of antisera were assessed by dot blotting with the synthetic peptides corresponding to kinase, MmpL and AIG1 proteins along with total proteins of *E. histolytica* (Eh proteins) and *E. moshkovskii* (**B**; Em protein)

**Fig. 6:**
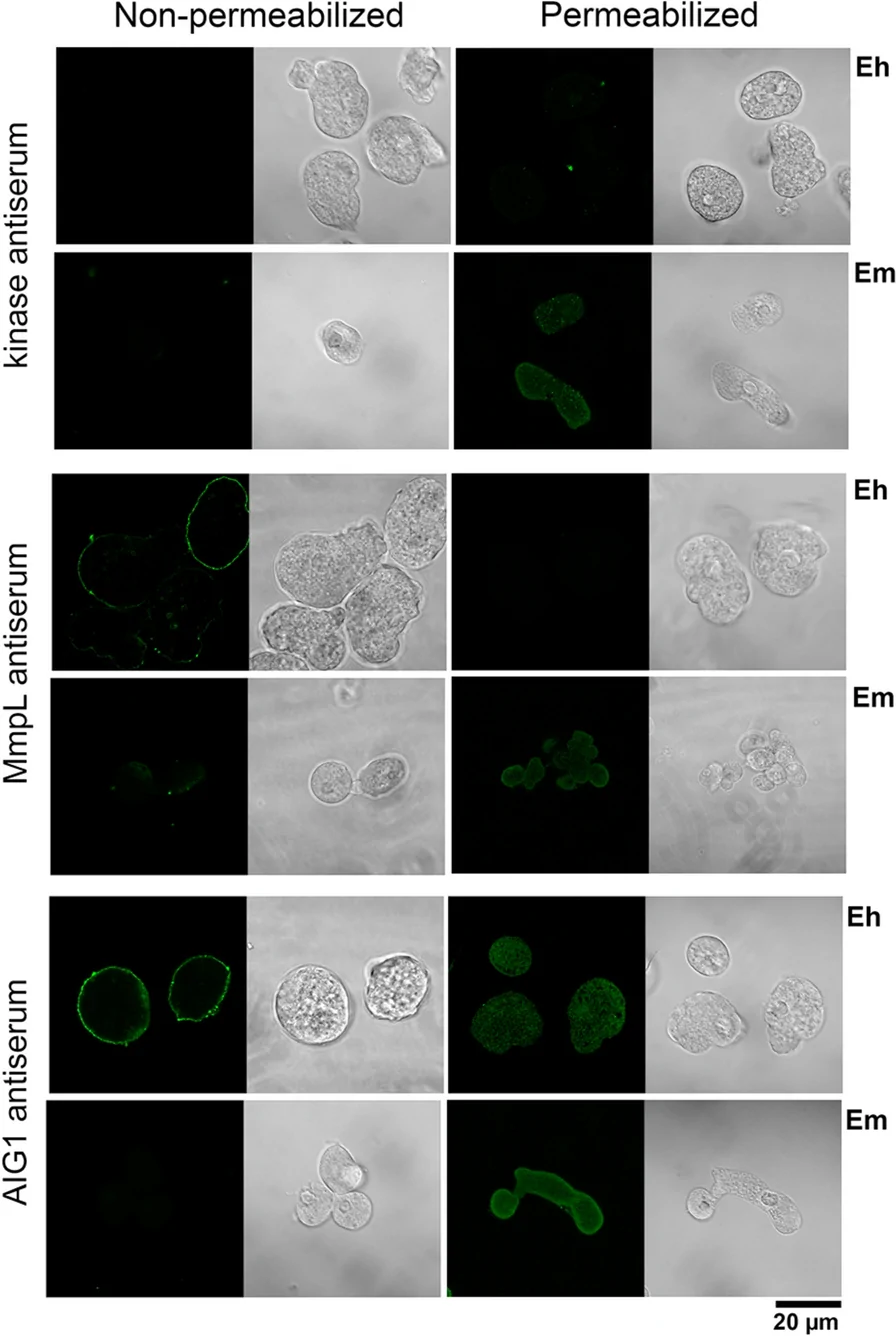
Cellular localization of GeLC–MS/MS identified proteins in the *E. histolytica* and *E. moshkovskii* trophozoites. Non-permeabilized and permeabilized cells of *E. histolytica* (Eh) and *E. moshkovskii* (Em) were reacted with the mouse serum after six-dose immunization with *Entamoeba* proteins and synthetic peptides of kinase, MmpL and AIG1-family protein (AIG1). Bound antibodies were tracked and visualized with FITC-labeled anti-mouse immunoglobulins

Three proteins were selected based on criteria mentioned in the Methods to validate protein expression and subcellular localization using peptide-specific antisera. Protein searching of the sequence ENTVLTNK showed high identity with hypothetical proteins (EHI_174880, EMO_071290) using AmoebaDB. BLASTp showed that the peptide matched to predicted lipid transporter or Mycobacterial membrane protein large; MmpL (COG2409) or ActII transport protein (TIGR00833). The peptide NKNDNYF was matched with hypothetical proteins of *E. histolytica* and *E. moshkovskii* (EHI_073610, EMO_084260). While BLASTp showed about 86% of the peptide identity with putative protein serine/threonine kinase of *E. invadens* IP1 (*E* value = 0.21 and 100% query coverage). The sequence MQEEGGINLE was highly identical to putative AIG1 protein (EHI_119040, EMO_058760). BLASTp showed 100% identity (*E* value = 0.30, 70% query coverage) with putative AIG1-family protein of *E. histolytica* HM-1: IMSS (XP_648471.1) and *E. histolyt*ica KU27(EMD43712.1). ELISA and dot blot assays confirmed the presence of these proteins in trophozoite-cell lysates (Fig. 5A, B). Immunofluorescent staining revealed distinct subcellular localization patterns of the MmpL and AIG1 proteins in *Entamoeba* trophozoites. MmpL and AIG1-family proteins localized at the outer membrane in *E. histolytica*, while these proteins were found predominantly at the inner membrane or within intracellular compartments of *E. moshkovskii*. The kinase localized intracellularly of *E. moshkovskii* trophozoites (Fig. 6).

## Discussion

*E. moshkovskii* possesses a genome with 1,594 orthologous genes within 126 gene family, while *E. histolytica* contains 8,306 genes distributed across 6,501 family [6]. Our comprehensive comparative proteomic analysis offers molecular and cellular insights that differentiate an invasive *Entamoeba histolytica* from non-invasive *E. moshkovskii* trophozoites. The identification of over 1,000 proteins per species, with 801 shared proteins and distinct sets unique to each, underscores the complexity and diversity of the robust *Entamoeba* proteome. Although the overall proteome sizes are comparable, approximately 30% of *E. moshkovskii* remain uncharacterized. This significant proportion of uncharacterized proteins underscores current gaps in knowledge and signals a promising direction for future functional investigations. Although the dataset was representative of three-independent biological preparations but a single technical LC–MS/MS run. The single LC–MS/MS acquisition may limit the ability to estimate technical variance and to apply more robust statistical tests. Aligned with study’s aim, we present differential protein expression here as exploratory, hypothesis generating results. We acknowledge the single LC–MS/MS acquisition may limit protein identification confidence and reproducibility. Future studies employing multiple biological and technical replicates, multiple testing correction, missing value imputation, and advanced visualizations will enhance reproducibility and proteomic impact.

Of the proteins with known functions, 73% are common in both species. Shared proteins are enriched with kinases, GTPase-activating proteins, and heat shock proteins, representing essential cellular machinery required for survival, environmental adaptation, and stress response in both *Entamoeba* species [11, 16, 26]. By contrast, previous reports have highlighted that kinases and GTPases also contribute in cytoskeletal dynamics, vesicular transport, and stress response that promote pathogenicity of *E. histolytica* [27, 28]. Differences in membranous abundance of Rho GTPase and Hsp70 [29] as well as compositions and complexity of kinase families, may influence the virulence of *Entamoeba* [30]. BspA, an outer membrane protein of *Bacteroides forsythus*, binds to fibronectin and fibrinogen, mediating bacterial colonization at the oral gravity. In amoebic colitis, *E. histolytica* trophozoites adhere to fibronectin and alter actin polymerization, facilitating trophozoite invasion across colon submucosa [2, 31]. *E. histolytica* trophozoite contains leucine-rich repeat containing BspA-like proteins, similar to non-pathogenic *E. dispar*, but with unique leucine-rich repeats with the conserved cysteine residues [32]. These observations suggest that gut environmental factors, such as the microbiota and host immunity, may influence *Entamoeba* pathogenicity and clinical outcomes. Our proteomic analysis initially annotated 15 and 5 of leucine-rich repeat containing proteins in *E. histolytica* and *E. moshkovskii*, respectively, but only five BspA proteins were identified from *E. moshkovskii* after GO re-classification in 2026*.* This may reflect the lack of unique BspA sequences or the presence of many hypothetical and uncharacterized proteins, highlighting the need for improved *Entamoeba* annotation resources and targeted functional genomics research.

Despite conserved of many proteins, our findings reveal distinct proteomic signatures that shed light on the divergent biology and pathogenicity of these species*. E. histolytica* displays a pronounced expression of proteins involved in nitrogen compound metabolism and vesicle-mediated transport, as well as a diverse array of catalytic activities. These results are consistent with reports linking metabolic versatility and enhanced vesicular trafficking to enhance virulence and tissue invasion in pathogenic strain of *E. histolytica* [10, 12]. The high abundance of the 70-kDa heat shock protein, phospholipid-translocating P-type ATPase, and AIG1-family proteins is notable, as analogous proteins have been implicated in stress tolerance, membrane remodeling, and immune evasion in other protozoa [6, 33]. In contrast, *E. moshkovskii* shows higher expression of the proteins associated environmental adaptation such as actin, signal recognition particle receptors and enzymes involved in lipid and small molecule metabolism. This proteomic profile indicates that *E. moshkovskii* is well-equipped for metabolic flexibility and resilience under changing in environmental conditions, in line with its frequent detection in asymptomatic, non-invasive cases, presenting diarrhea, abdominal pain and colitis [34]. Abundance of proteins involved in protein glycosylation and mitochondrial function may enhance *E. moshkovskii* ability to survive outside the host or under environmental stress, suggesting its ecological distinction from *E. histolytica.*

Many of *Entamoeba* proteins potentially involved in adaptation or pathogenicity remain uncharacterized [6]. Integrating proteomic profiling with experimental validation enhances the reliability and biological significance of the proteomic findings [15]. By combining mass spectrometry data with peptide-specific antibody validation and immunolocalization, we established a practical workflow for experimentally validating select hypothetical proteins. This approach confirmed the expression and subcellular localization of candidate proteins kinase, AIG1-family and MmpL proteins, providing a foundation for future studies to functionally analyze of uncharacterized proteins [17]. While this platform delivers valuable reference data, focusing on axenic trophozoites may not fully represent the spectrum of protein expression during actual intestinal infection, especially in the presence of gut microbiota and host immune factors. Future research should incorporate in vivo or gut-mimicking infection models, such as co-cultivation of axenic trophozoites with human colonic epithelial cell lines and representative gut microbiota. Additionally, functional studies employing gene silencing and targeted characterization of specific proteins will be critical to elucidate their precise roles and biological relevance with pathogenesis and host–parasite interactions.

AIG1-family protein has been identified as a novel virulence of *E. histolytica*, contributing in tissue invasion and amoebic liver abscess formation, immune escape through increased-parasite resistance to complement-mediated lysis [35]. Our proteomic findings demonstrated outer membrane localization of AIG-1 protein and MmpL in *E. histolytica,* while these proteins were found at the inner membrane or intracellular in *E. moshkovskii*. Mycobacterial MmpL proteins act as lipid exporters and contribute in multiple functions [36], including formation of cell envelope and virulent lipids, regulation of macrophage function, and host immune activation [37, 38]. Such differences in protein localization may help explain the distinct pathogenic properties and host immune responses of these *Entamoeba* species [17, 39].

Importantly, the prominent and species-specific subcellular locations of AIG1-family and MmpL proteins position them as promising targets for therapeutic intervention, studies of host–parasite interactions and immune responses, and diagnostic discovery. Despite high abundance of kinase in *E. histolytica*, its subcellular localization was not detected, possibly due to the use of polyclonal antibodies against de novo peptides with high identity to *E. invadens* kinase, and reflecting the high diversity among kinase sequences in *E. histolytica* compared to *E. moshkovskii* and *E. invadens.*

## Conclusions

Our comparative proteomic analysis provides a foundational resource for dissecting the molecular determinants of invasiveness of *E. histolytica* and environmental adaptation of *E. moshkovskii*. Proteins MmpL and AIG1, residing at the outer membrane of *E. histolytica* is proposed as potential targets for differential diagnosis between *E. histolytica* and *E. moshkovskii*. We also established the approach to integrate proteomics with experimental validation for the discovery of new diagnostics and therapeutic targets. These findings not only aid in the understanding of parasite biology, but also lay the groundwork for future investigation on development of targeted diagnostics, therapeutics, and interventions for amoebic infections.

## Supplementary Information

Additional file 1: Table S1. Datasets obtained from mass spectrometry were processed, quantified using DeCyder-MS software and protein identified by protein searching using Mascot across NCBI as mentioned in Methods section.

Additional file 2: Table S2. Peptides that are common in *E. histolytica* and *E. moshkovskii *were searched for description or annotated proteins by AmoebaDB.

Additional file 3: Table S3. Proteins presented in *E. histolytica* dataset were calculated for their relative expressions and presented as log_2_. Symbols – and + indicate decreased or increased in a protein expression in comparison to the *E. moshkovskii * trophozoite.

Additional file 4: Table S4. Proteins presented in *E. moshkovskii *dataset were calculated for their relative expressions and presented as log_2_. Symbols – and + indicate decreased or increased in a protein expression in comparison to the *E. histolytica* trophozoite.

## Data Availability

The datasets supporting the conclusions of this article are included within the article and its additional files.
